# Vaccination with a combination of planktonic and biofilm virulence factors confers protection against carbapenem-resistant *Acinetobacter baumannii* strains

**DOI:** 10.1038/s41598-022-24163-z

**Published:** 2022-11-19

**Authors:** Fatemeh Ramezanalizadeh, Iraj Rasooli, Parviz Owlia, Shakiba Darvish Alipour Astaneh, Raziyeh Abdolhamidi

**Affiliations:** 1 Department of Biology, Shahed University, Tehran, Iran; 2 Department of Biology, Molecular Microbiology Research Center, Shahed University, Tehran-Qom Express Way, Tehran, 3319118651 Iran; 3 Department of Biotechnology, Semnan University, Central Administration of Semnan University, Campus 1, P.O. Box 35131-19111, Semnan, Islamic Republic of Iran

**Keywords:** Immunology, Microbiology

## Abstract

*Acinetobacter baumannii* is a multi-drug resistant pathogen with the ability to switch between planktonic and biofilm phenotypes. Although there is no vaccine against *A. baumannii* infections, many attempts have been made to develop vaccines using planktonic or biofilm antigens. To cover the different phenotypes of *A. baumannii* during growth and attachment, we combined planktonic upregulated antigens of iron receptors with biofilm upregulated antigens of pilus rods and evaluated immune responses and protective efficacies of the combined vaccine using lethal and sub-lethal murine sepsis models. The results showed that the combined vaccine elicited high IgG antibody titers and conferred protection against lethal doses of two Carbapenem-resistant high adherent *A. baumannii* strains. Complete bacterial clearance from all the affected tissues of the mice challenged with *A. baumannii* was an excellent achievement with our quadrivalent immunogen. These results demonstrate both planktonic and biofilm antigens are important during antigen selection for vaccine design.

## Introduction

During the COVID-19 pandemic, studies have shown ESKAPE pathogens (*Enterococcus faecium*, *Staphylococcus aureus*, *Klebsiella pneumoniae*, *Acinetobacter baumannii*, *Pseudomonas aeruginosa*, and *Enterobacter* spp.) have severely affected the treatment of patients admitted in ICUs^1–3^. Multidrug-resistant *A. baumannii* was one of the most frequent pathogens isolated from patients hospitalized in the ICU for severe COVID-19 pneumonia^4^ or bacteremia^5^. In 2021, the World Health Organization (WHO) introduced antibacterial vaccines as an effective tool to reduce antibiotic resistance^6^. Recombinant vaccines based on protein antigens are good options for combating infections caused by *A. baumannii*; earlier studies have also confirmed the protective potential of these proteins. There is a serious problem in designing recombinant vaccines against *A. baumannii*, neglecting that *A. baumannii*, like other nosocomial pathogens, has different lifestyles, including planktonic, pellicle biofilm, and surface-attached biofilm^7^. Today, transcriptomic and proteomic studies have demonstrated that virulence genes expressed, and proteins produced in different lifestyles of *A. baumannii* are distinct^8–11^, so vaccine candidates selected solely based on planktonic mode may fail to influence biofilm mode and vice versa. Results of a recent study have highlighted the importance of planktonic and biofilm phenotypes during antigen selection for vaccine design against biofilm-forming *S. aureus*^12^. As a result, the selection of virulence factors with high levels of expression, playing roles in different lifestyles of the pathogen is of prime importance.

Iron receptors related to multiple uptake pathways of *A. baumannii* planktonic cells are virulence factors that highly up-regulate in the host environment^13^. Six different gene clusters for active iron uptake have been identified among different strains of *A. baumannii*: (i) ferrous iron uptake cluster; (ii) hemT and hemO clusters (heme uptake), and (iii) baumannoferrins, acinetobactin, and fimsbactins clusters (synthesis and transport of the siderophore)^14^. Among them, acinetobactin cluster is the most important iron cluster in most strains, which uses the BauA transporter for iron uptake. A recent study showed that gene *bauA* is essential for bacterial pathogenesis during a murine sepsis model of *A. baumannii* ATCC17978^15^. Acinetobactin siderophore scavenge iron from transferrin and lactoferrin, non-heme iron binding glycoproteins which store extracellular ferric iron. Heme is the greatest reservoir of iron in the human host and is critical for functions including oxygen transport, enzymatic reactions, cellular respiration, mitochondrial energy production, antioxidant defense, and signal transduction^16,17^. *A. baumannii* strains can capture iron-bound heme via two specialized heme uptake systems, hem T and/or hemO^18^. *A. baumannii* ATCC19606 has a hemT uptake system. Zimbler et al.^19^ showed that *A. baumannii* ATCC 19606 strain defective in the *bauA* gene (BauA-) can grow on heme as an iron source under iron-restricted conditions mediated by the addition of iron chelator 2,2′-dipyridyl, suggesting the importance of hemT cluster in iron metabolism.

The chaperone–usher (CU) pili are important virulence factors that up-regulate in biofilm styles (pellicle and surface-attached). Biofilm is crucial for the spreading and persistence of an infection in the host^20^. CU pili are encoded as single operons and organized in four-gene clusters encoding a fimbrial adhesin (a flexible end tip) that enable bacteria to interact with biotic or abiotic surfaces, a major pilus subunit (a rigid long rod), an outer membrane usher pore protein involved in pili assembly, a periplasmic chaperone that involved in folding pilus subunits and targeting them to the usher^21,22^. Components of pili located on the surface of the bacterium are immunogenic and represent an attractive target for vaccine development. Initially, Langermann et al.^23,24^ prepared the FimCH vaccine, a subunit vaccine made based on the end tip region of type I pili, FimH, and its cognate chaperone, FimCH showed significant protection against uropathogenic *Escherichia coli* (UPEC) challenge in subcutaneously vaccinated mice and intramuscular vaccinated cynomolgus monkey. Recently, studies showed that the pilus rods play essential roles in the pathogenesis of gram-negative bacteria due to the unique properties of their spring-like structure^25,26^. Spaulding et al.^25^ showed that the pilus rod of Enterotoxigenic *E. coli* (ETEC) is necessary for host–pathogen interactions and to promote bacterial colonization in different sites of infection. Seo et al.^27^ showed that antibodies induced by pilus rod subunit and tip adhesion subunit equivalently inhibit bacterial adherence in vitro and suggested that different pili subunits, rod or tip adhesion, are equally effective in inducing neutralizing anti-adhesion antibodies. To date, four different types I chaperone-usher gene clusters have been identified in *A. baumannii* strains: (i) A1S_2213-2218 (Csu-cluster), (ii) AB57_2003-2007 (p pili), (iii) A1S_1507-1510, and (iv) A1S_2088-2091^28^. Major subunits of Csu-cluster (CsuA/B) and p pili cluster (FimA) have been predicted as vaccine candidates based on reverse vaccinology and proteomic approaches^29^. CsuA/B and FimA are pilus rods that up-regulate in pellicle and biofilm styles^30,31^.

We have already reported that immunization of mice with bivalent vaccines composed of BauA-HemTR or CsuA/B-FimA antigens induce a high titer of antibodies in mice after vaccination and that partial protection was observed after challenge with a lethal dose of *A. baumannii*^32,33^. Since there is a variation in up/down-regulation in type I chaperone-usher pili and siderophore clusters depending on the different lifestyles of *A. baumannii*, the combination of four proteins can probably cover a wide range of growth modes. In this work, we have generated recombinant CsuA/B, FimA, BauA, and HemTR proteins and evaluated the protective effect of the quadrivalent vaccine against two highly adhesive strains of *A. baumannii*.

## Results

### Selection of *A. baumannii* clinical strains for animal experiments

**Figure 1:**
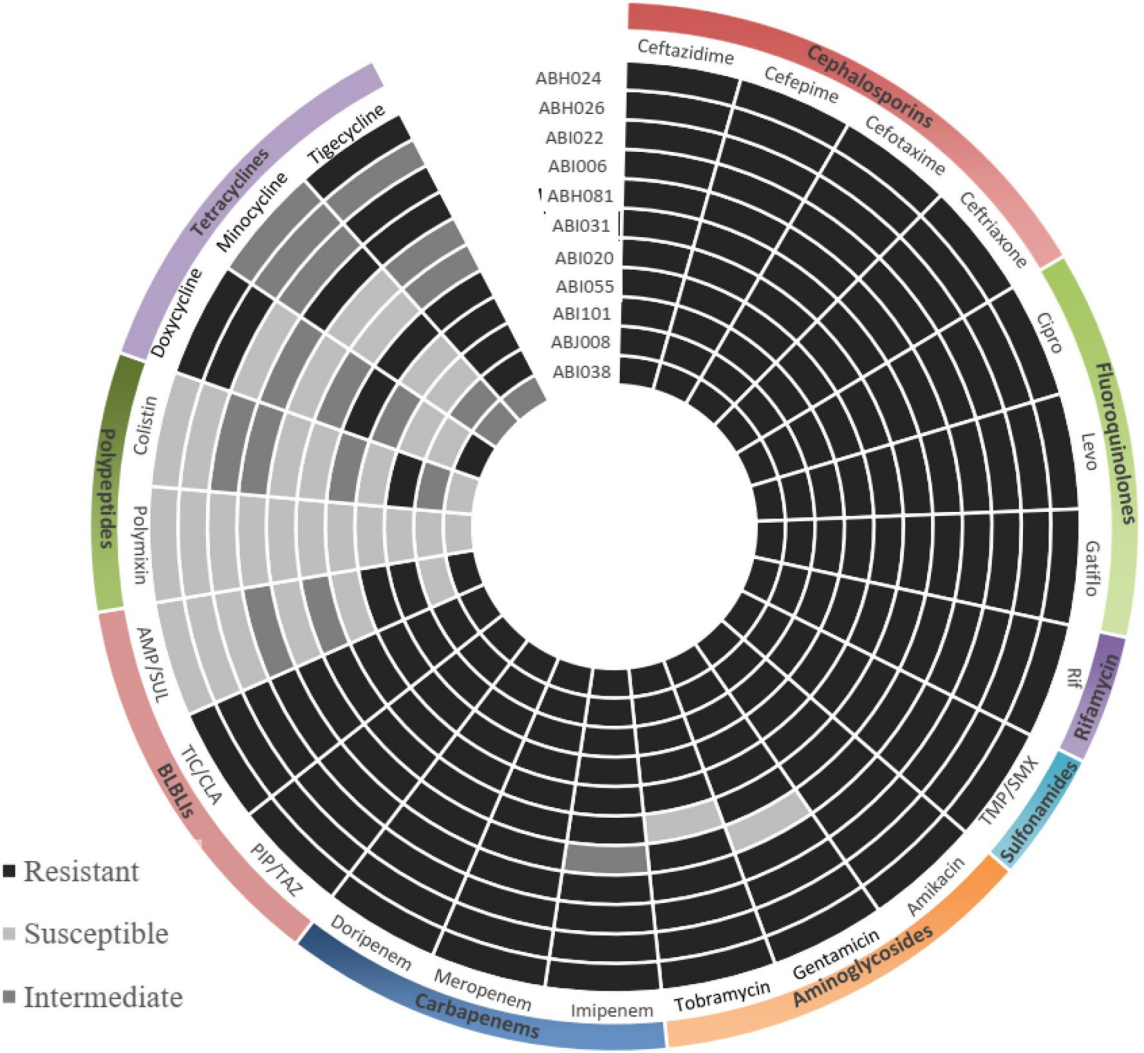
Drug resistance pattern of clinical isolates of *A. baumannii*. Drug resistance of eleven clinical isolates of *A. baumannii* is presented. Among these multi-drug resistance strains, ABI022 and ABI038 were selected for animal studies based on adherence assays to biotic and abiotic surfaces. *Cipro* ciprofloxacin, *Levo* Levofloxacin, *Rif* Rifampicin, *TMP/SMX* Trimethoprim/sulfamethoxazole, *BLBLIs* β-lactam-β-lactamase inhibitor antibiotics, *PIP/TAZ* Piperacillin/tazobactam, *TIC/CLA* Ticarcillin/clavulanate, *AMP/SUL* Ampicillin/sulbactam.

**Figure 2:**
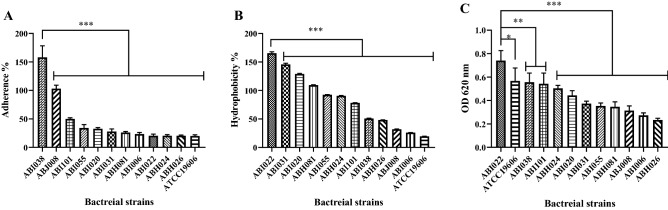
Adherence to A549, hydrophobicity, and biofilm formation assays results for clinical isolates of *Acinetobacter baumannii*. (**A**) A549 monolayer cells (10^5^ cell/mL) were incubated with *A. baumannii* isolates (10^6^ CFU/mL) for 3 h. ABI038 isolate was significantly more adherent than other isolates and *A. baumannii* ATCC19606 (control strain). (**B**) Cell surface hydrophobicity of isolates was measured using Microbial Adhesion to Hydrocarbon. Adherence to p-xylene by ABI022 isolate was significantly different the adherence by other clinical isolate and control strain. (**C**) Quantitative analysis of biofilm formation by *A. baumannii* isolates was determined by a microtiter-plate assay. The clinical ABI022 formed a higher biofilm in comparison with other clinical isolates (*p* < 0.01, *p* < 0.001) and *A. baumannii* ATCC19606 (*p* < 0.05). Data are shown as mean ± SD and statistical differences determined by one-way ANOVA and Dunnett's correction (***p < 0.001, **p < 0.01, * *p* < 0.05).

In order to determine if the quadrivalent vaccine proposed in this study can be effective against Carbapenem-resistant high adherent *A. baumannii* strains, two screening steps were performed. In the first step, among 43 clinical strains of *A. baumannii*, 11 isolates were selected based on an antimicrobial susceptibility test. These strains were resistant to cephalosporins, fluoroquinolones, rifamycin, sulfonamides, aminoglycosides, and carbapenems (Fig. 1). The adherence, hydrophobicity, and biofilm formation of 11CRAB strains were evaluated in the second screening. In the cell adherence assay, ABI038 showed the highest adhesion to A549 cell lines (Fig. 2A). In the hydrophobicity and biofilm assays, ABI022 showed the greatest hydrophobicity (Fig. 2B) and produced more biofilm than other isolates (Fig. 2C). The ABI022 and ABI038 clinical strains were selected for animal studies.

### Detection of the genes in clinical strains

PCR was used to detect genes encoding CsuA/B, FimA, BauA, and HemTR proteins in both clinical strains. The products were analyzed by gel electrophoresis. The full length of CsuA/B, FimA, BauA, and HemTR coding sequences of *A. baumannii* were 543, 534, 2130, and 2670 bp, respectively. Except for the *hemTR*, all the genes (*csuA/B*, *fimA*, and *bauA*) were detected in both clinical strains of *A. baumannii*. The *hemTR* gene was found only in ABI038.

### Expression and purification of proteins

**Figure 3:**
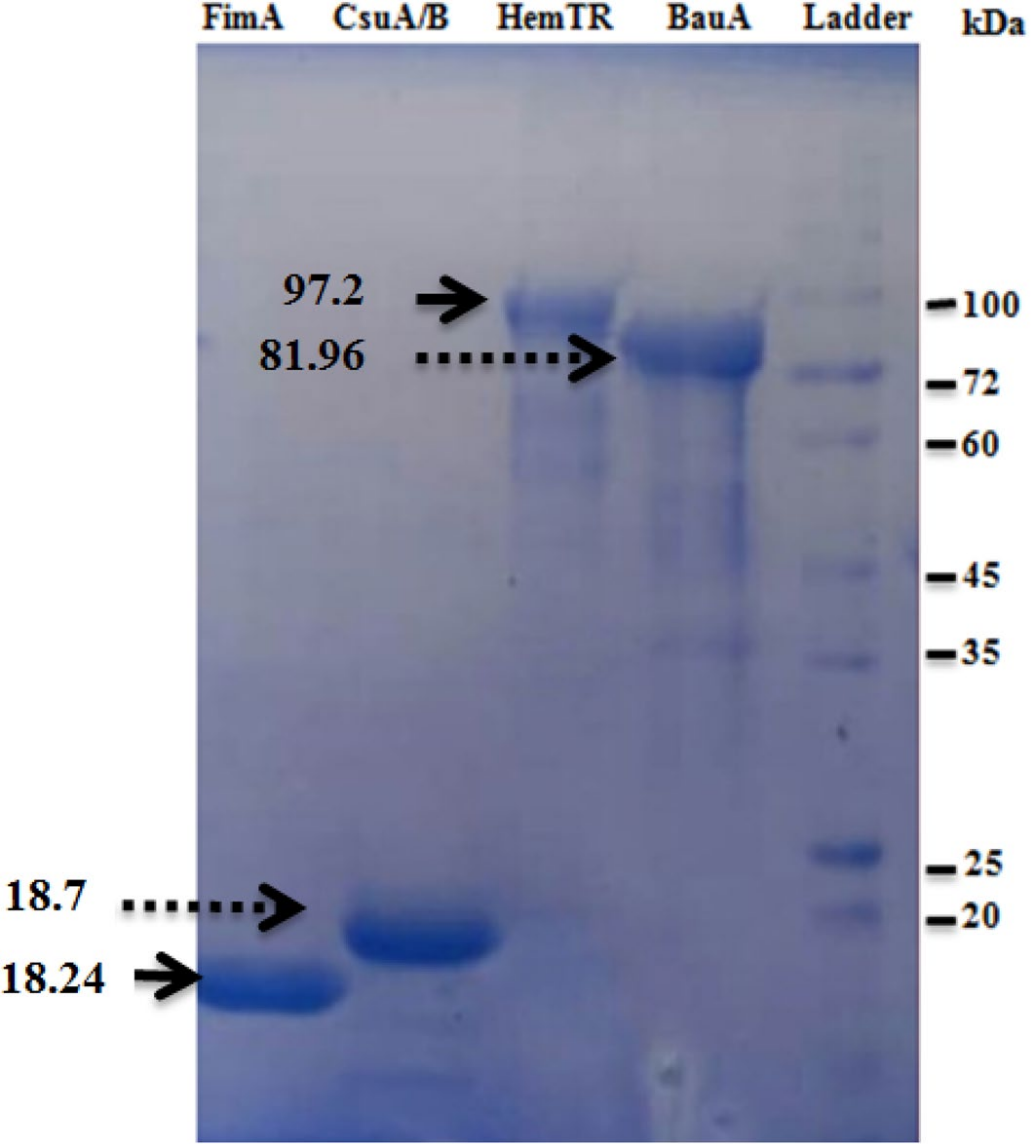
Sodium dodecyl sulfate–polyacrylamide gel electrophoresis (12%) analysis of recombinant proteins. Recombinant proteins containing His-tagged residues were expressed in *E. coli* BL21 (DE3), followed by purification by affinity chromatography using an Ni–NTA agarose matrix. Purity was evaluated by SDS-PAGE and Coomassie blue staining.

All four recombinant proteins (CsuA/B, FimA, HemTR, and BauA) expressed successfully in *E. coli* BL21cells. SDS-PAGE analysis indicated that the recombinant proteins were present as insoluble inclusion bodies. Buffers containing 8–6 M urea were used for the solubilization of the inclusion bodies. Solubilized proteins were purified by Ni–NTA chromatography,and refolded by urea gradient dialysis. As mentioned in our previous study^32,33^, due to precipitations proteins at decreasing the concentrations of urea from 2 M to PBS, arginine (0.2–0.5 M) was added to the refolding buffer as an inhibitor. Refolding was successful because antibodies elicited against refolded recombinant proteins can react with *A. baumannii* cells in a whole-cell ELISA test, suggesting recognition of epitopes on native proteins. Proteins were resolved on SDS-PAGE and visualized by Coomassie Brilliant Blue staining. The molecular weights of recombinant proteins were predicted as 18.7, 18.24, 97.2, and 81.96 kDa for CsuA/B, FimA, HemTR, and BauA, respectively, using the ProtParam tool. The molecular weights of proteins on SDS-PAGE gel were approximately corresponding to their predicted size (Fig. 3).

### Antigen-specific IgG responses generated by CsuA/B-FimA-BauA-HemTR combination

**Figure 4:**
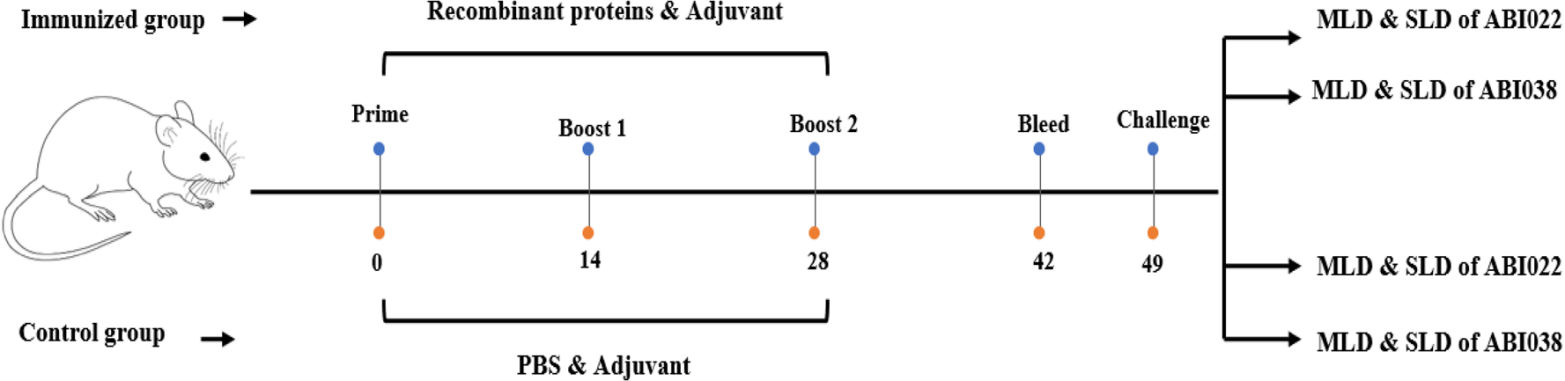
Flow chart of immunization and challenge schedules. Mice received three immunization doses with the 40 µg of combination of proteins (10 µg of FimA + 10 µg of CsuA/B + 10 µg of HemTR + 10 µg of BauA/each) mixed with adjuvants at two weeks intervals. Antibody responses were measured using blood sera from mice on day 42. Mice were challenged with MLD and SLD of *A. baumannii* strains on day 49.

**Figure 5:**
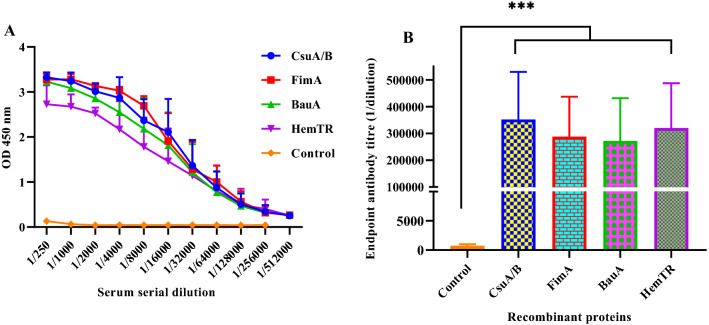
Total IgG responses after vaccination with the combination of CsuA/B-FimA-BauA-HemTR. (**A**) Data display the absorbance values (OD_450_) at two-fold serial dilutions of the sera collected two weeks after the last immunization, specific responses to each recombinant protein were determined by coating of antigens separately in 96-well plates. (**B**) The sera from four mice of each group (test and control) were tested in duplicate for analyzing IgG titers responses against the immunizing proteins. Significant (*p* values) between the control and recombinant proteins of the test group were determined by a Kruskal–Wallis test followed by Dunn’s multiple comparisons (*p* < 0.05).

To evaluate the immunogenicity of recombinant proteins, BALB/c mice had divided into two groups which subcutaneously were immunized three times with the combination of four proteins (immunized group) or PBS (control group) at a 14-day interval (Fig. 4). Mouse sera (n = 4) were collected on day 42, 2 weeks after the last immunization, to evaluate antigen-specific antibody responses using the ELISA test. The result showed that anti-pilus and anti-receptor total IgG levels elevated so high that the absorbance value (OD 450) was observed even at the highest dilution (1:256,000) (Fig. 5A). Antibody titer levels in the immune group were significantly higher than in the control (*p* < 0.001). Notably, the level of IgG raised to pilus proteins was similar to the level of anti-iron receptor antibody, and no significant difference was noted between them (Fig. 5B).

### Determination of minimal lethal and sub-lethal doses of *A. baumannii* strains

**Figure 6:**
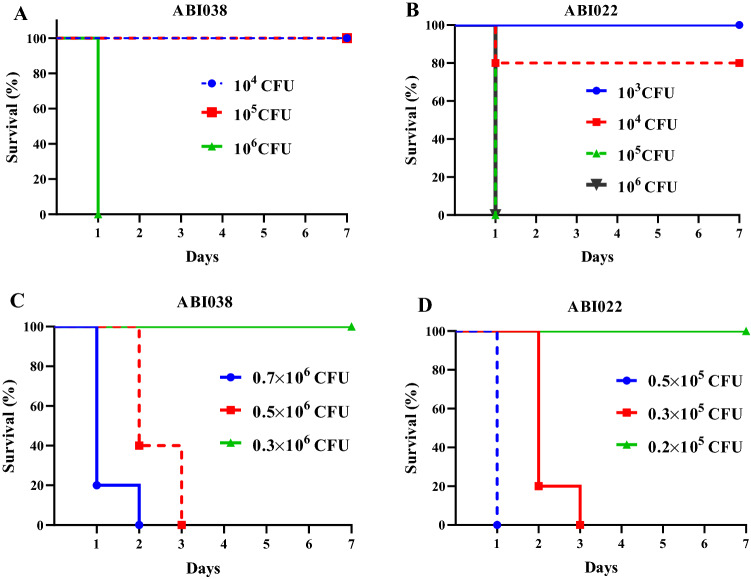
Determination of minimal lethal dose (MLD) and sub-lethal dose (SLD) of clinical isolates. (**A**,**B**) The lethal dose of clinical strains of ABI038 and ABI022 were 10 ^6^ and 10^5^, respectively. (**C**) MLD and SLD of ABI038 were determined as 0.7 × 10^6^ and 0.5 × 10^6^, respectively. (**D**) MLD and SLD of ABI022 were determined as 0.5 × 10^5^ and 0.3 × 10^5^, respectively.

To confirm the effectiveness of the quadrivalent vaccine in a sepsis model, it is necessary to determine the degree of virulence of each clinical strain. The mice were administered with intraperitoneal injection of 0.5 mL of different doses of *A. baumannii* strains admixed with mucin at final doses of 10^3^, 10^4^, 10^5^, and 10^6^ CFU per animal. Survival was monitored for 7 days. Survival rates in mice infected with ABI038 were 100, 100, 100, and 0%, respectively. Survival rates in the more virulent strain, ABI022, were 100, 80, 0, and 0%, respectively (Fig. 6A,B). The bacterial dose of 10^6^ CFU was selected as the lethal dose for *A. baumannii* ABI038 (Fig. 6A). The lethal dose for ABI022 was 10^5^ CFU (Fig. 6B). In the second step, the minimal lethal (MLD) and sub-lethal (SLD) doses of each strain were determined by reducing the bacterial concentrations of the lethal dose. The dose of 0.7 × 10^6^ CFU of the ABI038 and 0.5 × 10^5^ CFU of ABI022 were determined as the MLD, whereas 0.5 × 10^6^ CFU of the strain ABI038 and 0.3 × 10^5^ CFU of the strain ABI022 were considered as the SLD (Fig. 6C,D).

### Bacterial challenge

**Figure 7:**
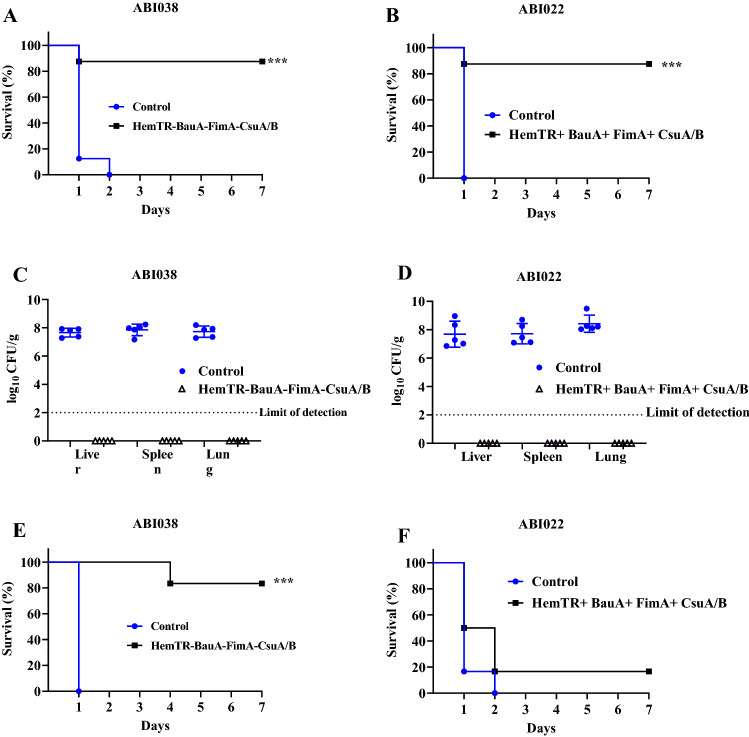
Active and passive immunization against MDR *A. baumannii* clinical isolates ABI038 and ABI022. (**A**,**B**) Active immunization with a combination of HemTR-BauA-FimA-CsuA/B recombinant proteins protected mice from minimal lethal dose of clinical isolates. The mice (n = eight mice per group) monitored for 7 days. (**C**,**D**) Bacterial burden was high in all organs of the adjuvant treated control mice, no colonies were detected after 24 h of infection in test groups challenged with sub-lethal dose of ABI038 and ABI038 (n = five mice per group). (**E**,**F)** Passive immunization with HemTR-BauA-FimA-CsuA/B antibodies protected mice (n = six mice per group) from minimal lethal dose of ABI038, survival rate was 16.66% in mice challenged with ABI022. ***p < 0.001 compared to survival in control mice.

The minimal-lethal and sub-lethal sepsis models were used to evaluate the immuno-protectivity of the recombinant proteins against the challenge with *A. baumannii* clinical strains ABI038 and ABI022. In the MLD challenge, the survival rate of the immunized mice against both strains was similar, and one of eight mice died on day one. 87.5% of immunized mice survived over seven days of monitoring. The differences were significant between the immunized and control groups (*p* < 0.001) (Fig. 7A,B). The effect of the immunogen was further confirmed in SLD sepsis where no colonies were detected in the spleen, lungs, and liver samples after 24 h, while 7–8 log CFU/g was determined in all the 3 organs of the control groups challenged with ABI022 or ABI038 (Fig. 7C,D). In the passive immunization, immune sera conferred protection against a lethal dose of ABI038 (83.3% survival), and a significant (*p* < 0.001) difference was observed between the control and immune group (Fig. 7E). The challenge with the ABI022 isolate resulted in poor protection of 50% (Fig. 7F).

Since epitopes in antigenic surface proteins tend to mutate to evade the humoral response of the host, the determination of epitope conservation of these proteins was necessary. We previously demonstrated that B-cell epitopes of CsuA/B and FimA are highly conserved among 20 *A. baumannii* strain isolated from bacteremia, sputum, wound, blood, and osteomyelitis samples^32^. Accordingly, in this study, a similar method was used. The results showed that approximately 30% of B cell epitopes of BauA have more than 70% conservancy. In the HemTR protein, the conservation was higher, approximately 50% of B cell epitopes had a conservancy greater than 70% (Supplementary Tables ST-1, ST-2, and ST-3).

## Discussion

*Acinetobacter baumannii* has three different lifestyles of planktonic, pellicle biofilm, and surface-attached biofilm cells^7^. Treating infections caused by carbapenem-resistant *A. baumannii* (CRAB) is challenging because they are resistant to most antibiotics^34^. In addition, biofilm formation and adherence to biotic and abiotic surfaces create an additional barrier to treatment^35^. Because of the variable expression of antigens among these styles, the selection of vaccine candidates against *A. baumannii* is a challenge. In a previous study, we selected *A. baumannii* biofilm-associated pilus proteins, CsuA/B and FimA, as vaccine candidates and evaluated their protective efficacy against *A. baumannii* at lethal and sub-lethal doses in murine sepsis models. The results showed that the combination of pilus proteins could confer partial protection^32^. A possible reason for partial protection is the down-regulation of the *A. baumannii* type I pili genes in the animal host. Murray et al.^13^ showed that *csuA/B* and *csuA-E* (A1S_2213-8) genes were strongly down-regulated in mice, a mammalian host, in an early step of bacteremia. An in vitro study also has shown that parts of the *csu* operon like the *csuA/B* gene are down-regulated under iron starvation^36^. Expectedly, iron uptake genes highly have increased in both studies (in vitro, in vivo)^13,36^. It seems iron uptake systems are more important than adhesion factors like type I pili upon entering bacteria into the host due to nutritional immunity and iron starvation. *A. baumannii* can scavenge iron directly from heme and siderophores using TonB-dependent receptors such as BauA and hemTR. BauA (receptor related to acinetobactin cluster) and HemTR (receptor related to hemT cluster) were predicted as vaccine candidates among different strains of *A. baumannii* by reverse vaccinology^33,37^. Specifically, our group evaluated the immuno-protective efficacy of these siderophore-heme receptor proteins against *A. baumannii* in lethal and sub-lethal murine sepsis models. Our finding showed that receptors confer partial protection in a lethal challenge^33^. One possible explanation for this observation also returns to the regulation of gene expression. A comparative proteomic study showed that the levels of expression of iron proteins in immature pellicle (1-day) are higher than of mature pellicle (4-day) while CsuA/B proteins were abundant in mature pellicle^8^, suggesting pili type I proteins are more important than iron proteins in pellicle mode. Similar result was also observed in a comparative transcriptomic study between surface-attached biofilm and planktonic cells of *A. baumannii*. The expression of *bauA* gene was six-fold higher in biofilm cells than in planktonic, while the expression of *csuA/B* gene was 164-fold in biofilm cells, as compared with planktonic cultures^11^. This seesaw-like movement between type I pili and iron uptake genes is probably an attempt to rapid adaptation from the planktonic to biofilm phenotype, a hypothesis that must be tested. In this study, we used the combination of biofilm (CsuA/B, FimA) and planktonic (BauA, HemTR) derived antigens for protection against the sepsis model of *A. baumannii*. Surprisingly, antibody titers against each of the four antigens were significantly increased (mean > 256,000) after the last immunization. No significant differences were observed between anti-pili and anti-iron receptor antibody titers, suggesting that pili and iron receptors contribute equally to elicitation of immune responses in the mammalian hosts.

Planktonic bacteria adhere to biotic or abiotic surfaces and form biofilm. If highly adhesive bacterial strains possess the greatest potential for switching from planktonic to a sessile mode in response to environmental conditions, we used two high adherent *A. baumannii* strains for animal studies. ABI022 and ABI038 strains were isolated from wound and tracheal secretions of hospitalized patients in a medical intensive care unit (ICU), respectively. Both strains were resistant to cephalosporins, fluoroquinolones, sulfonamides, aminoglycosides, and even carbapenems. Carbapenem-resistant *A. baumannii* (CRAB) strains are a global and critical priority according to the WHO and threatened public health^38^. Virulence of these strains was checked by intraperitoneal injection of different doses (10^3^–10^6^ CFU) of ABI022 and ABI038 mixed with mucin in mice. The ABI038 strain exhibited the highest level of adhesion to the A549 cells. However, biofilm formation and surface hydrophobicity were higher in the ABI022 strain. ABI022 was found to be more virulent (tenfold) than ABI038. This sepsis model was used to investigate the protective abilities of a quadrivalent vaccine against two CRAB strains. Active immunization effectively protected mice from the lethal and sub-lethal challenges of ABI022 and ABI038 strains, as determined by better survival of the immunized mice (87.5%) and suppressed bacterial burdens in all tissues. Compared to our previous studies where Csu-FimA and HemTR- BauA antigens were studied as bivalent immunogens in a similar sepsis model^32,33^, the quadrivalent vaccine was a better formulation for preventing *A. baumannii* infections.

Mellata et al.^39^ conducted a similar study using a quadrivalent vaccine composed of common pilus antigens EcpA and EcpD and iron uptake proteins IutA and IroN in *E. coli*, which led to effective protection against *E. coli* sepsis. Furthermore, they showed that antibodies raised against four antigens were effective in passive immunization, especially when combined with anti-poly-*N*-acetylglucosamine (PNAG) antibodies. PNAG is a major component of the biofilm matrix of pathogens, including *E. coli*, *A. baumannii*, and *S. aureus*^40,41^. Our results in passive immunization were strain dependent. Protection in mice challenged with ABI038 was higher than ABI022, i.e., 83.3% versus 16.66%. A plausible reason for this finding may be to the variations in the surface structures of the two strains. Wang-Lin et al.^42^ showed that the capsular polysaccharide of *A. baumannii* AB307-0294 impedes antibody binding to the specific targets. The ABI022 strain may have a thick capsule that impedes the binding of anti-Csu-Fim-BauA-HemTR antibodies in passive immunization. However, this reason is questionable because the capsule is intrinsically a hydrophilic polymer and ABI022 is a strain with high surface hydrophobicity. This paradox was reported in the recently published studies where some pathogenic fungal strains that form a biofilm on medical devices manifest high surface hydrophobicity despite the presence of the hydrophilic capsule^43,44^. Three different types of vaccines can be designed based on antigens produced by bacteria in a biofilm: (1) the vaccines that target antigens located inside the bacteria cell (bacterial-cell antigens) including pili, flagellin, and Bap; (2) the vaccines that target antigens exported to the matrix (biofilm-matrix-derived antigens) including PNAG, DNA, and extracellular proteins; (3) those that target mixed antigens, bacterial-cell and biofilm-matrix-derived antigens. Fattahian et al.^45^ targeted Bap, a bacterial cell antigen, and observed high protection against *A. baumannii* Kh0060 strain in a sepsis model of mice. Bentancor et al.^46^ targeted PNAG in a high-PNAG-producing strain of *A. baumannii* and demonstrated that anti-PNAG antibodies have protective efficacy in bacteremia and pneumonia models of BALB/c mice. Although these studies confirm the importance of biofilm antigens in protection against *A. baumannii*, vaccines prepared solely based on biofilm antigens may not provide full protection against planktonic infections. A recent study showed that the pentavalent vaccine prepared by combining both planktonic and biofilm antigens is a better formulation for protection against *S. aureus* infections. The pentavalent vaccine had decreased mortality and increased bacterial clearance in comparison to the quadrivalent vaccine prepared only of biofilm antigens^12^. Our data demonstrate that the combination of pili and iron receptor proteins promotes protection against lethal doses of high adherent *A. baumannii* strains. It may be noted that we used a sepsis model and not in vivo models of biofilm-related infections such as tissue-related and device related-infections. Therefore, further investigations are needed to study the efficacy of our vaccine in other animal or medical device models.

Previously, we demonstrated that CsuA/B, FimA, BauA, and HemTR have conserved among 2927, 2300, 1965, and 456 strains of *A. baumannii* with identity more than 97%, respectively^32,33,47^. A recent study also showed the genes involved in the synthesis of CsuA/B and BauA are part of the core genome of each MDR isolate of *A. baumannii*^48^. As a result, immunization with these proteins likely targets many strains of *A. baumannii*. But genomic plasticity in *A. baumannii* creates problems in the development of a successful vaccine at the strain level. As seen in this study, *hemTR* was not in ABI022, and little protection against this isolate had observed in passive immunization. The subject notably was addressed in a pan-genome study of *A. baumannii*^49^. They showed that three classes of gene clusters of *A. baumannii* have variations in insertion or deletion among *A. baumannii* strains isolated from the military healthcare system viz (i) type I pili (role in adherence), (ii) siderophores (role in iron acquisition), and (iii) efflux pumps (role in efflux). Interestingly, most respiratory isolates had no gene cluster type I pili (csu deletions). The gain and loss of virulence gene content probably are due to rapid adaption to both abiotic and biotic environments where nutrients are scarce.

However, the quadrivalent vaccine proposed in this study can probably cover a wide range of strains than monovalent vaccines designed based on only a virulence factor. Polyvalent vaccines would target multiple mechanisms of virulence and different phenotypes of *A. baumannii*, such as adherence, biofilm formation, metabolism, and growth.

In conclusion, the results highlight the importance of both planktonic and biofilm phenotypes during antigen selection for vaccine design. The quadrivalent vaccine provided good protection against two CRAB strains. High bacterial clearances in all tissue of mice challenged with *A. baumannii* was an excellent achievement with our quadrivalent immunogen. Additionally, antibody titers against each protein were high after booster doses.

## Materials and methods

### Approval for animal experiments

All experimental protocols were approved by the Ethics Committee of Shahed University (Tehran, Iran). All methods were carried out in accordance with relevant guidelines and regulations of the National Institute of Health guide for the care and use of laboratory animals (NIH Publication No_8023, revised 1978). Six- to eight-week-old female BALB/c mice were purchased from the University of Tehran (Tehran, Iran) and housed in clean cages, and fed with a standard antibiotic-free diet and water ad libitum.

### Approval for human experiments

The project was approved by Ethics Committee of Shahed University (Tehran, Iran). The patients were not directly involved in the study. The diagnostic samples taken from hospitalized patients by medical experts were sent to pathology and microbiology laboratory of Mustafa Khomeini hospital (Tehran, Iran). We confirm that all methods involving human were carried out in accordance with relevant guidelines and regulations. In order to protect the privacy of patients, the data was de-identified. Therefore, informed consent was abandoned by the Ethics Committee of Shahed University (Tehran, Iran).

### Bacteria

Two screening steps were performed to select *A. baumannii* strains: (I) Forty-three strains of *A. baumannii* already isolated from the wounds (13 isolates), blood (15 isolates), and tracheal secretions (15 isolates) of patients hospitalized in various wards and intensive care units (ICUs) for diagnostic and treatment purposes, were used (Supplementary Table ST-2). The strains were identified using preliminary conventional phenotypic tests including growth on MacConkey agar, motility, sugar fermentation, catalase, and oxidase tests, and other standard recommended tests^50,51^.Confirmation of clinical isolates was performed by PCR amplification by the 16S-23S ribosomal DNA intergenic spacer region of *A. baumannii*^52^. Disc diffusion tests were used to determine antibiotic resistance. Breakpoints were determined according to the clinical and laboratory standards institute (CLSI) guidelines^53^. For antibiotics without established breakpoints by CLSI the following cut-offs were applied to define resistance: rifampicin > 2 mg/L (based on CLSI breakpoints for Staphylococci) and tigecycline > 2 mg/L (based on FDA breakpoint)^54^. (II) Strains resistant to antibiotics especially carbapenems were selected for comparison in cell adhesion, biofilm, and hydrophobicity assays. *A. baumannii* ATCC19606 was used as a control. Strains that had high adhesion properties to biotic or abiotic surfaces were selected for challenge assays.

### Adherence to A549 cells

Quantitative adherence assays were performed as described previously^55^ with minor modifications. Briefly, 10^5^ A549 cells were seeded into each well of a 24-well tissue culture plate and incubated for 24 h at 37 °C before use. Monolayers were washed twice with DMEM medium. Then 10^6^ bacteria were added to the wells (ratio of bacteria to cells, 10:1). Infected cells were incubated for 3 h at 37 °C in an atmosphere of 5% CO_2_. A549 cells were washed four times with phosphate-buffered saline and digested with trypsin for 1–2 min. Serially diluted cell lysates were plated onto LB agar and incubated overnight at 37 °C. Adherence is defined as the percentage of bacteria attached to the A549 cells compared with the inoculum used to infect monolayers.

### Biofilm assay

Biofilm formation by *A. baumannii* strains on 96-well polystyrene plates was performed by previously described protocols with minor modification^56^. Briefly, 20 µL of overnight cultures grown in LB broth were inoculated to wells a 96-well plate containing 180 µL of M9 biofilm medium. After incubating for 5 days at 37° C, the wells were washed 3 times with PBS, dried, and stained with 1% crystal violet for 15 min. Bound cells were quantified by the addition of ethanol-acetone (80:20, v/v) and measurement of the solubilized stain at an optical density of 620 nm using a microtiter plate reader.

### Hydrophobicity assays

Cell hydrophobicity was assessed using a standard microbial adhesion to hydrocarbon (MATH) test^56^. In brief, overnight cultures of the *A. baumannii* strains grown on LB broth were centrifuged at 5000 rpm and washed twice with PUM buffer (22.2 g/L K_2_HPO_4_·3H_2_O, 7.26 g/L KH_2_PO_4_, 1.8 g/L urea, 0.2 g/L, MgSO_4_·7H_2_O; pH 7.1) then resuspended in PUM buffer to an optical density at 600 nm of 1 (= OD original). The bacteria were then mixed with p-xylene and vortexed for 2 min. the suspensions were rested at room temperature for 15 min to allow for phase separation. The final OD600 nm of the aqueous partition was determined (= OD final). Cell surface hydrophobicity based on bacterial adherence to hydrocarbon was measured using the following formula: %Adherence = [1 − (OD final/OD original] × 100.

### Detection of *csuA/B*, *fimA*, *hemTR*, and *bauA* genes in *A. baumannii* strains

*Acinetobacter baumannii* ATCC19606, ABI022, and ABI038 genomic DNA, to be used as template DNA in PCR reactions, isolated using the Cetyltrimethyl Ammonium Bromide (CTAB) method. Amplification of genes was conducted using Taq polymerase and specific primers designed for cloning these genes in the pET 28a vector (Supplementary Table ST-4). PCR amplifications were carried out for 30 elongation cycles consisting of 3-steps: pre-denaturation, elongation cycles (denaturation, annealing, and extension), and Final extension. The parameters for the amplification cycles used in each PCR experiment are presented in (Supplementary Table ST-5).

### Determination of minimal lethal (MLD) and sub-lethal doses (SLD) of *A. baumannii* strains

A mouse sepsis model was established according to the protocol laid out by Harris et al.^57^ with some modifications. Briefly, bacterial cells were removed from overnight cultures of *A. baumannii* strains in LB agar plates using an inoculating loop and were re-suspended in 3 mL of 0.85% saline to obtain a uniform suspension and rested for 5 min to allow clumps to settle. Subsequently, the supernatant was added to 100 mL of LB to reach an OD_600_ of 0.1. LB medium was transferred to an incubator for bacterial growth and reached to mid-log phase based on a previously determined relation curve between optical density and colony-forming units (CFUs). Serial dilution was performed to prepare 10^4^ to 10^7^ CFU/mL of *A. baumannii*. Inocula were prepared by mixing 100 µL of each dose of bacterial suspensions containing 10^4^, 10^5^, 10^6^, and 10^7^ CFU/mL, with 250 µL of 10% porcine mucin (w/v) plus 150 µL of saline (total volume 500 µL). This constituted the total injectable volume of 500 µL containing 10^3^, 10^4^, 10^5^, and 10^6^ CFU each, respectively. Mice (six per group) were intraperitoneally injected and monitored for 7 days. Minimal lethal and sub-lethal doses were determined as described in our previous study^32^.

### Expression and purification of pilus rod and iron receptor proteins

The pET28a containing full length of *csuA/B, fimA*, *bauA,* and* hemTR* genes were transformed into *E. coli* BL21 (DE3) and plated on LB-kanamycin agar plate. A single colony from each fresh agar plate was transferred into 2 mL of LB + 1% glucose-containing kanamycin and incubated in a rotary shaker at 180 rpm at 37 °C for 8 h. A total of 100 μL of the inoculum culture was transferred into 200 mL of ZYP-5052 autoinduction medium and incubated overnight with shaking at 180 rpm. The resulting cultures were centrifuged (4000 rpm for 30 min), and the cells were suspended in TE lysis buffer (Tris–HCl 10 mM, EDTA 1 mM). The cells were lysed using glass beads and vortexing at 4 °C for 15 min with 5 min intervals on ice. The lysates were pelleted by centrifugation at 13,000 rpm for 20 min at 4 °C. The resulting pellets were solubilized in denaturing buffer (6–8 M Urea, 10 mM Tris–HCl, 100 mM NaH_2_PO_4_·2H_2_O). The solubilized inclusion bodies were centrifuged at 20,000 rpm for 40 min at 4 °C, and the supernatant containing recombinant proteins was collected. The purification was performed under denaturing conditions by affinity chromatography using Ni–NTA resin. All the eluted fractions were analyzed by SDS-PAGE. Finally, recombinant proteins were refolded by the urea gradient dialysis method, and the protein concentration was estimated by *Bradford's* method.

### Animal immunization

Mice were subcutaneously injected with 10 µg of each recombinant protein (CsuA/B + FimA + BauA + HemTR = Total 40 µg) or PBS (control group) mixed with complete Freund's Adjuvants (Sigma) on day 0 and with incomplete Freund's Adjuvants (Sigma) on days 14 and 28. Immune sera were collected two weeks after the last immunization (on day 42). For active immunization, the mice were intraperitoneally challenged with MLD and SLD of *A. baumannii* strains three weeks after the third immunization. In the lethal challenge, the survival rates were recorded over the next seven days. In the sub-lethal challenge, five mice from each were sacrificed 24 h post-infection. The liver, lungs, and spleen were aseptically excised and homogenized in 2 mL of saline. The bacterial burden was determined in the tissue homogenates by plating serial dilutions of the homogenates on LB plates and incubating at 37 °C overnight. For passive immunization, 100 µL of decomplemented antisera collected on day 42 from the immunized mice were administered intravenously 3 h before the bacterial challenge with the MLD of *A. baumannii* strains. The sera from mice receiving only adjuvant served as the control.

### Serum antibody titers

Specific IgG titers in serum were measured by ELISA. Briefly, 5 μg/well of each refolded protein (CsuA/B, FimA, BauA, and HemTR) in sodium bicarbonate buffer (pH 9.6) were coated on the 96 well flat-bottom plates and incubated overnight at 4 °C overnight. The wells were washed three times with PBST and blocked using 5% skim milk (w/v) in PBST. After adding diluted sera to the wells, antibody binding was detected with the horseradish peroxidase-conjugated goat anti-mouse IgG diluted in PBST (1:15,000). The endpoint titer was defined as the highest dilution at which the optical density was 0.1 greater than that of the wells receiving control adjuvant sera.

### Statistical analysis

All statistical analyses were performed using GraphPad Prism 8.0 software. The data were presented as mean with standard deviations represented as error bars. The differences between adherence, hydrophobicity, and biofilm formation of clinical strains were determined by one-way ANOVA and Dunnett's correction test. A comparison of antibody titers was performed using a Kruskal–Wallis test followed by Dunn’s multiple comparison test. Non-parametric Log Rank test was used to compare the survival rate. Differences were considered significant if the *p*-value was < 0.05. (*p < 0.05; **p < 0.01; ***p < 0.001).

### Ethics approval

We confirm that this study is reported in accordance with ARRIVE guidelines. The principles stated in the Guide for the Care and Use of Laboratory Animals were followed. The animal care protocol was approved by the ethics committee of Shahed University.

## Supplementary Information

Supplementary Information.

## Data Availability

The datasets generated and/or analyzed during the current study are available from the corresponding author on reasonable request.
