# Supplementary material for: Vaccination with a combination of planktonic and biofilm virulence factors confers protection against carbapenem-resistant Acinetobacter baumannii strains
Source: Sci Rep. 2022 Nov 19;12:19909. doi: 10.1038/s41598-022-24163-z (PMC9675771; doi:10.1038/s41598-022-24163-z)
Supplement: Supplementary file 1 — Supplementary Information. [file 41598_2022_24163_MOESM1_ESM.docx]

| **B**  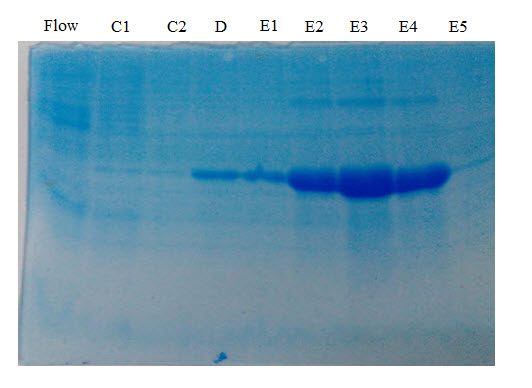  **18.24 kDa kDa** | **A**  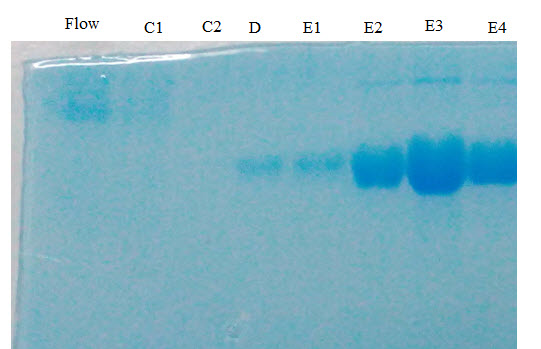  **18.7 kDa** |
| --- | --- |
| **D**  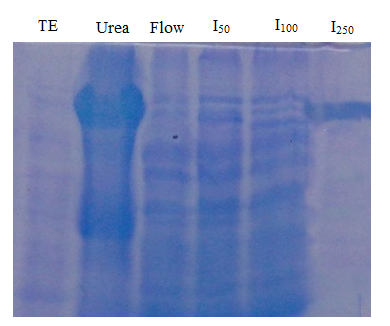  **81.96 kDa** | **C**  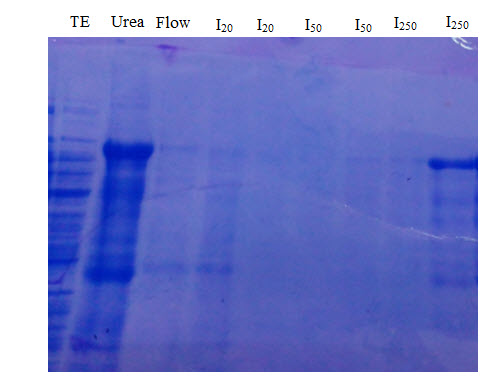  **97.2 kDa** |

**Figure S1: Expression and purification of recombinant proteins, (A) CsuA/B, (B) FimA, (C) HemTR, (D) BauA**

**Table ST-1: List of 20 *A. baumannii* strains used for assessing epitopes conservancy**

| **Number** | **Strain** | **Source of isolation/disease** | **Status** |
| --- | --- | --- | --- |
| 1 | AB307_0294 | Blood | Complete |
| 2 | AB0057 | Bloodstream | Complete |
| 3 | MDR-TJ | Hospital setting | Complete |
| 4 | BJAB07104 | Blood | Complete |
| 5 | BJAB0715 | Spinal fluid | Complete |
| 6 | BJAB0868 | Ascites | Complete |
| 7 | TYTH-1 | Bacteremia | Complete |
| 8 | D1279779 | Bacteremia | Complete |
| 9 | ATCC-17978 | Meningitis | Complete |
| 10 | TCDC-AB0715 | Bloodstream infection | Complete |
| 11 | LAC4 | Outbreak | Complete |
| 12 | 1656–2 | Sputum | Complete |
| 13 | ACICU | Cerebrospinal fluid | Complete |
| 14 | AC30 | Endotracheal secretion | Complete |
| 15 | DU202 | Clinical isolate | Complete |
| 16 | ZW85-1 | Diarrheal patient feces | Complete |
| 17 | ABNIH1 | Outbreak infection | Drafts |
| 18 | ABNIH3 | Outbreak infection | Drafts |
| 19 | NAVAL-18 | Wound | Drafts |
| 20 | AB5075 | Osteomylitis | Drafts |

**Table ST-2: List of 43 *A. baumannii* strains isolated from hospitalized patients**

| **Strain** | **Source** | **Strain** | **Source** | **Strain** | **Source** |
| --- | --- | --- | --- | --- | --- |
| ABH001 | Blood | ABH011 | Wound | ABH019 | Tracheal secretions |
| ABH033 | Blood | ABH012 | Wound | ABH020 | Tracheal secretions |
| ABI017 | Blood | ABH071 | Wound | ABH021 | Tracheal secretions |
| ABH080 | Blood | ABI018 | Wound | ABH022 | Tracheal secretions |
| ABH081 | Blood | ABI019 | Wound | ABH023 | Tracheal secretions |
| ABI001 | Blood | ABI022 | Wound | ABH024 | Tracheal secretions |
| ABI002 | Blood | ABH016 | Wound | ABJ008 | Tracheal secretions |
| ABI003 | Blood | ABI025 | Wound | ABH026 | Tracheal secretions |
| ABI039 | Blood | ABH018 | Wound | ABI055 | Tracheal secretions |
| ABI040 | Blood | ABI008 | Wound | ABI024 | Tracheal secretions |
| ABJ012 | Blood | ABI032 | Wound | ABI101 | Tracheal secretions |
| ABJ013 | Blood | ABI015 | Wound | ABI038 | Tracheal secretions |
| ABJ009 | Blood | ABI045 | Wound | ABI006 | Tracheal secretions |
| ABJ010 | Blood |  |  | ABI031 | Tracheal secretions |
| ABJ011 | Blood |  |  | ABI020 | Tracheal secretions |

**Table ST-3: Characterizations of epitopes**

| **Epitope #** | **Epitope name** | **Epitope sequence** | **Epitope length** | **Percent of protein sequence matches at identity <= 100%** | **Minimum identity** | **Maximum identity** |
| --- | --- | --- | --- | --- | --- | --- |
| 15 | 15 | VSYNRNFKTGNDQRINHRSF | 20 | **100.00% (20/20)** | 90.00% | 100.00% |
| 26 | 26 | IEDKQAKDITEVIAATDPSI | 20 | **100.00% (20/20)** | 80.00% | 100.00% |
| 29 | 29 | MFGRVEVLKGPSALLNGMPP | 20 | **100.00% (20/20)** | 80.00% | 100.00% |
| 31 | 31 | QQTAQTNVAALPAITVKAEQ | 20 | **100.00% (20/20)** | 90.00% | 100.00% |
| 34 | 34 | FVLNGVASAQMALRLGYALG | 20 | **100.00% (20/20)** | 95.00% | 100.00% |
| 41 | 41 | FGGHVDVGRRFGENKEFGVR | 20 | **100.00% (20/20)** | 85.00% | 100.00% |
| 48 | 48 | VFVLCASNTYAAVIDNSTKT | 20 | **100.00% (20/20)** | 90.00% | 100.00% |
| 50 | 50 | PFNTISYTDKYIEDKQAKDI | 20 | **100.00% (20/20)** | 80.00% | 100.00% |
| 55 | 55 | AALPAITVKAEQDDTYAGGQ | 20 | **100.00% (20/20)** | 95.00% | 100.00% |
| 12 | 12 | MSMNGLFGITPFYRTSPEMF | 20 | **85.00% (17/20)** | 70.00% | 100.00% |
| 14 | 14 | FARLTTTYMSDAQFGGHVDV | 20 | **85.00% (17/20)** | 70.00% | 100.00% |
| 16 | 16 | NYYIRGYASSTNDMSMNGLF | 20 | **85.00% (17/20)** | 30.00% | 100.00% |
| 22 | 22 | KATDKISVYANYIEGLTKGD | 20 | **85.00% (17/20)** | 65.00% | 100.00% |
| 28 | 28 | SALLNGMPPAGSVGGTVNLV | 20 | **85.00% (17/20)** | 75.00% | 100.00% |
| 35 | 35 | SIYTNGASGGWSENYYIRGY | 20 | **85.00% (17/20)** | 50.00% | 100.00% |
| 42 | 42 | QPQLTNLALGAPRTYMLSVS | 20 | **85.00% (17/20)** | 65.00% | 100.00% |
| 43 | 43 | RGIEWSFFGSPIEHVRLMGG | 20 | **85.00% (17/20)** | 60.00% | 100.00% |
| 32 | 32 | VIDNSTKTLEQQTAQTNVAA | 20 | **70.00% (14/20)** | 65.00% | 95.00% |
| 1 | 1 | FDVDKKSADAGFKGKFETGS | 20 | **30.00% (6/20)** | 50.00% | 95.00% |
| 2 | 2 | GFLGSKKFLDTPFNTISYTD | 20 | **30.00% (6/20)** | 65.00% | 100.00% |
| 3 | 3 | TSSTGTLSSTLGQLAFDVDK | 20 | **30.00% (6/20)** | 30.00% | 100.00% |
| 4 | 4 | VNVSTAVGIPKPPKADTLLS | 20 | **30.00% (6/20)** | 40.00% | 100.00% |
| 5 | 5 | EIFPPQKTKQQELGLKVDLG | 20 | **30.00% (6/20)** | 60.00% | 100.00% |
| 6 | 6 | PFYRTSPEMFGRVEVLKGPS | 20 | **30.00% (6/20)** | 75.00% | 100.00% |
| 7 | 7 | SVPGRTLLDVGARYSTKVED | 20 | **30.00% (6/20)** | 55.00% | 100.00% |
| 8 | 8 | YINAENTLSVPGRTLLDVGA | 20 | **30.00% (6/20)** | 50.00% | 100.00% |
| 9 | 9 | TKQQELGLKVDLGTFAHTLS | 20 | **30.00% (6/20)** | 55.00% | 100.00% |
| 10 | 10 | QAPATASNPGEIFPPQKTKQ | 20 | **30.00% (6/20)** | 45.00% | 100.00% |
| 11 | 11 | AGSVGGTVNLVTKYAADEPF | 20 | **30.00% (6/20)** | 55.00% | 100.00% |
| 13 | 13 | DYGYRIIPGFSDPVITNIYD | 20 | **30.00% (6/20)** | 30.00% | 100.00% |
| 17 | 17 | KPPKADTLLSPDWGSVETKD | 20 | **30.00% (6/20)** | 50.00% | 100.00% |
| 18 | 18 | ARVFVDAYDALDHVDGVTRG | 20 | **30.00% (6/20)** | 50.00% | 100.00% |
| 19 | 19 | LGLDWQGENARVFVDAYDAL | 20 | **30.00% (6/20)** | 50.00% | 95.00% |
| 20 | 20 | LTNKAYWAQPQLTNLALGAP | 20 | **30.00% (6/20)** | 70.00% | 100.00% |
| 21 | 21 | TFRANIYNLTNKAYWAQPQL | 20 | **30.00% (6/20)** | 65.00% | 100.00% |
| 23 | 23 | VANATYYNHTQDDYGYRIIP | 20 | **30.00% (6/20)** | 40.00% | 95.00% |
| 24 | 24 | STLGQLAFDVDKKSADAGFK | 20 | **30.00% (6/20)** | 35.00% | 100.00% |
| 25 | 25 | DHVDGVTRGVNVSTAVGIPK | 20 | **30.00% (6/20)** | 45.00% | 100.00% |
| 27 | 27 | QVATSSNVGFLGSKKFLDTP | 20 | **30.00% (6/20)** | 55.00% | 100.00% |
| 30 | 30 | WDTQVAQGTLTLSGNINAVS | 20 | **30.00% (6/20)** | 35.00% | 100.00% |
| 33 | 33 | TLPENAKSATTPGVALLIKA | 20 | **30.00% (6/20)** | 40.00% | 100.00% |
| 36 | 36 | GTLTLSGNINAVSKQYINAE | 20 | **30.00% (6/20)** | 50.00% | 100.00% |
| 37 | 37 | NKEFGVRINGMYRDGDAAVN | 20 | **30.00% (6/20)** | 60.00% | 100.00% |
| 38 | 38 | GEYDFSDQLMAYAAYGQSTT | 20 | **30.00% (6/20)** | 60.00% | 100.00% |
| 39 | 39 | QAKLGAEWDTQVAQGTLTLS | 20 | **30.00% (6/20)** | 40.00% | 100.00% |
| 40 | 40 | DGHTAVAVPKNQAKLGAEWD | 20 | **30.00% (6/20)** | 55.00% | 100.00% |
| 44 | 44 | YNGASAGTITSSTGTLSSTL | 20 | **30.00% (6/20)** | 25.00% | 100.00% |
| 45 | 45 | LADTLSFAQDKVQLTLGLRH | 20 | **30.00% (6/20)** | 70.00% | 100.00% |
| 46 | 46 | DWGSVETKDKGAMIRGEYDF | 20 | **30.00% (6/20)** | 55.00% | 100.00% |
| 47 | 47 | PFLFHSTLSTSSFGLADTLS | 20 | **30.00% (6/20)** | 40.00% | 100.00% |
| 49 | 49 | QLMAYAAYGQSTTEYKYNGA | 20 | **30.00% (6/20)** | 55.00% | 100.00% |
| 51 | 51 | LTKTKSGGNDGHTAVAVPKN | 20 | **30.00% (6/20)** | 35.00% | 100.00% |
| 52 | 52 | YDPNPNWGPKPEFTPPFLFH | 20 | **30.00% (6/20)** | 25.00% | 100.00% |
| 53 | 53 | FTYLDPELTKTKSGGNDGHT | 20 | **30.00% (6/20)** | 40.00% | 100.00% |
| 54 | 54 | GMYRDGDAAVNDQSKESRLF | 20 | **30.00% (6/20)** | 40.00% | 100.00% |
| 56 | 56 | EHVRLMGGFTYLDPELTKTK | 20 | **30.00% (6/20)** | 55.00% | 100.00% |
| 57 | 57 | TKPSSYLDPSKLVNNLPTFV | 20 | **30.00% (6/20)** | 25.00% | 100.00% |
| 58 | 58 | FSDPVITNIYDPNPNWGPKP | 20 | **30.00% (6/20)** | 30.00% | 100.00% |
| 59 | 59 | SKESRLFSLGLDWQGENARV | 20 | **30.00% (6/20)** | 55.00% | 95.00% |
| 60 | 60 | LKVDLGTFAHTLSAFEITKP | 20 | **30.00% (6/20)** | 40.00% | 100.00% |
| 61 | 61 | QSTTEYKYNGASAGTITSST | 20 | **30.00% (6/20)** | 35.00% | 100.00% |
| 62 | 62 | NLPTFVSDGEQRNRGIEWSF | 20 | **30.00% (6/20)** | 55.00% | 100.00% |

**Table ST-4. The primer sequences used in PCR assays for pilus rod and iron receptor genes detection in *A. baumannii* strains**

| Gene | Primer | Nucleotide sequence (5' → 3'; enzyme sites in bold faces) | Amplicon size |
| --- | --- | --- | --- |
| *csuA/B* | forward  reverse | AGCA**GGATCC**GCTGTTACTGGTCAGGTTGACG  CCGG**GCGGCCGC**TTAGAAATTTACAGTGACTA | 543 bp |
| *fimA* | forward  reverse | AGCT**GGATCC**GCTGATGGTACAATTACAATTAATGG  CCGG**GTCGAC**TTGATAGATAATTGTGTATTGAACAGAAG | 534 bp |
| *hemTR* | forward  reverse | AGCA**GGATCC**GCAGAAACGGAACAATCAAGTACC  CCAT**GCGGCCGC**GAACTTATATGTACCTCGTAC | 2670 bp |
| *bauA* | forward  reverse | GAGCC**GGATCC**GCTGTTATTGATAATTCAACAAAAAC  CGCG**CTCGAG**AAAGTCATATGATACAGATAGCATATACG | 2131 bp |

**Table ST-5. The PCR amplification program used for the pilus rod and iron receptor genes**

| **Amplification of *hemTR*** | | **Amplification of *bauA*** | | **Amplification of *fimA*** | | **Amplification of *csuA/B*** | | **Step** | |
| --- | --- | --- | --- | --- | --- | --- | --- | --- | --- |
| **Time** | **Temp. (°C)** | **Time** | **Temp.**  **(°C)** | **Time** | **Temp. (°C)** | **Time** | **Temp. (°C)** |  |  |
| 5 min | 94 | 5 min | 94 | 4 min | 94 | 4 min | 94 | **Initial denaturation** | |
| 1 min | 94 | 1 min | 94 | 30 s | 94 | 30 s | 94 | **Denaturation** | **35 cycles** |
| 49 s | 58.3 | 40 s | 65 | 45 s | 66.1 | 45 s | 66.9 | **Annealing** |  |
| 2.5 min | 72 | 2.20 min | 72 | 45 s | 72 | 45 s | 72 | **Extension** |  |
| 5 min | 72 | 5 min | 72 | 5 min | 72 | 5 min | 72 | **Final extension** | |
